# Supplementary material for: Neuropathy-causing TRPV4 mutations disrupt TRPV4-RhoA interactions and impair neurite extension
Source: Nat Commun. 2021 Mar 4;12:1444. doi: 10.1038/s41467-021-21699-y (PMC7933254; doi:10.1038/s41467-021-21699-y)
Supplement: Supplementary file 1 — Supplementary Information [file 41467_2021_21699_MOESM1_ESM.pdf]

Supplementary Figure 1

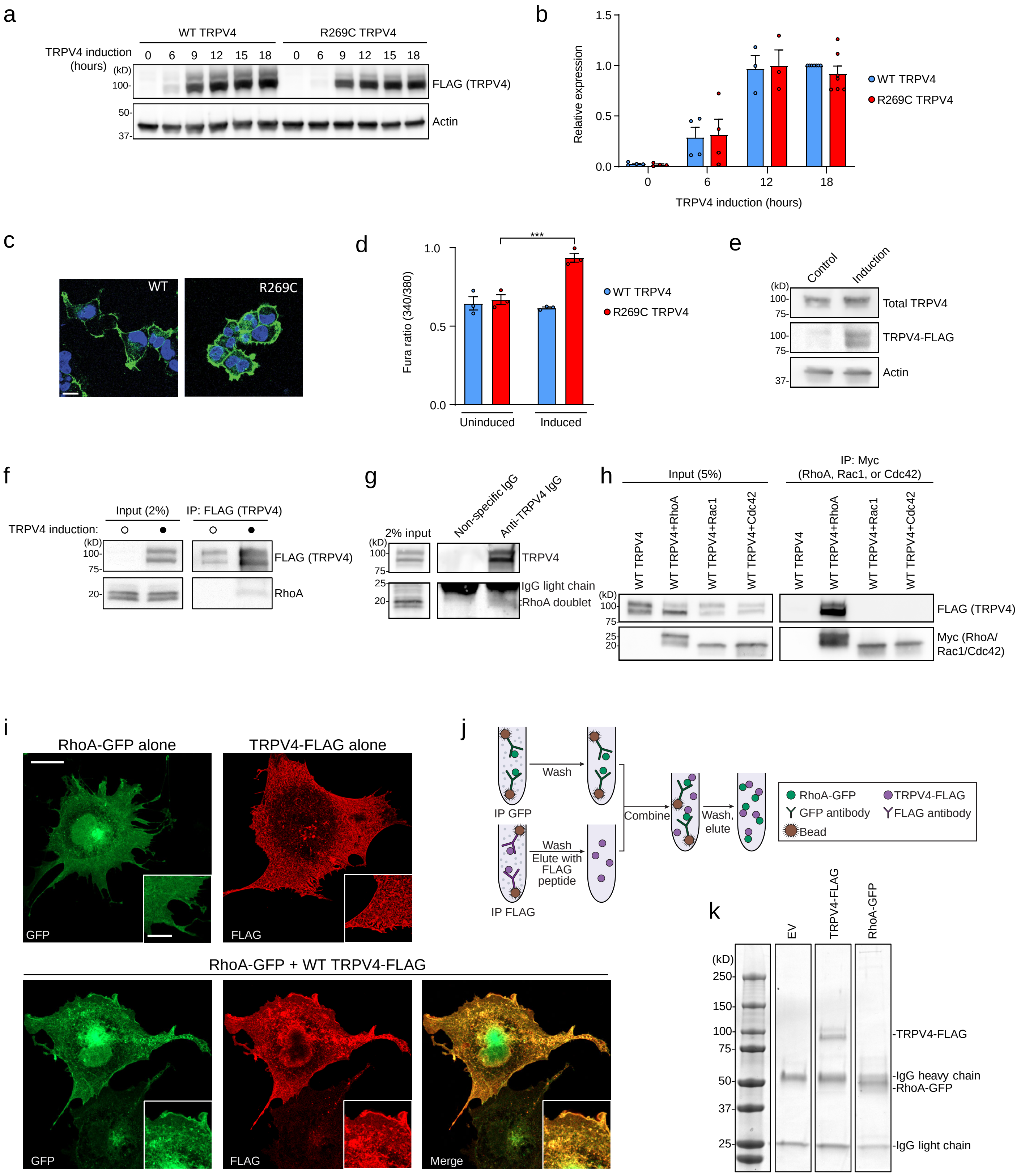

Supplementary Figure 1. TRPV4-RhoA interactions are specific

(a-b) T-Rex-TRPV4^{WT} or T-Rex-TRPV4^{R269C} cells were either left untreated or induced with tetracycline (15 ng/ml) for 6, 9, 12, 15, or 18 h followed by western blot with anti-FLAG antibody (a) and quantification of TRPV4 band intensity normalized to WT TRPV4 at 18 h (b), n = 4 (0 h), 3 (6 h), 3 (12 h), and 7 (18 h) independent experiments. TRPV4-FLAG expression is robustly induced with tetracycline with low background expression.

(c) Immunofluorescence images of T-Rex-TRPV4^{WT} or T-Rex-TRPV4^{R269C} cells induced with tetracycline (15 ng/ml) demonstrates enrichment of TRPV4 at the plasma membrane. Scale bar, 10 µm. Representative image from three independent experiments.

(d) T-Rex-TRPV4^{WT} or T-Rex-TRPV4^{R269C} cells were either left untreated or induced with tetracycline (15 ng/ml) for 18 h followed by assessment of basal calcium levels by calcium imaging using Fura-2 AM. Expression of R269C TRPV4 leads to increased basal calcium levels, consistent with gain of ion channel function. One-way ANOVA, followed by Tukey's multiple comparisons test, n = 3 independent experiments, ***p = 0.0006.

(e) T-Rex-TRPV4^{WT} cells were either left untreated or induced with tetracycline (15 ng/ml) for 18 h followed by western blot to assess levels of total TRPV4 and TRPV4-FLAG. TRPV4-FLAG induction results in a modest increase in total TRPV4. Representative blot from at least three independent experiments.

(f) T-Rex-TRPV4^{WT} cells were treated with tetracycline or untreated, then subjected to immunoprecipitation with anti-FLAG antibody. Endogenous RhoA co-immunoprecipitates with TRPV4 only with induction of TRPV4 expression. Presence of TRPV4-FLAG in uninduced immunoprecipitation lanes is due to low-level expression leak. Representative blot from at least five independent experiments.

(g) Mouse choroid plexus was dissected from fresh brain tissue and subjected to immunoprecipitation with anti-TRPV4 antibody, followed by western blot for TRPV4 and RhoA. Endogenous TRPV4 co-immunoprecipitates endogenous RhoA. Blot from one independent experiment.

(h) MN-1 cells were transfected with TRPV4-FLAG and RhoA-Myc, Rac1-Myc, or Cdc42-Myc and then subjected to immunoprecipitation with anti-Myc antibody. TRPV4 co-immunoprecipitates with RhoA, but not Rac1 or Cdc42. Representative blot from two independent experiments.

(i) Confocal immunofluorescence images of COS7 cells transfected with TRPV4-FLAG and RhoA-GFP alone or in combination demonstrates co-localization of TRPV4 and RhoA at the plasma membrane and within the cytoplasm. Representative image from two independent experiments. Scale bar, 20 µm and 10 µm for inset.

(j) Schematic representation of the approach utilized for the co-incubation experiment shown in Figure 1e. Immunopurified TRPV4-FLAG and RhoA-GFP were prepared from separate cell lysates followed by several washes. TRPV4-FLAG was then eluted from the bead-antibody complexes and co-incubated with bound, immunopurified RhoA-GFP followed by several washes.

(k) Coomassie staining of immunopurified TRPV4-FLAG and RhoA-GFP demonstrates enrichment for TRPV4 and RhoA with minimal presence of additional proteins. Images are of non-contiguous lanes from the same gel. Representative image from two independent experiments.

Data are presented as mean values +/- SEM.

Supplementary Figure 2

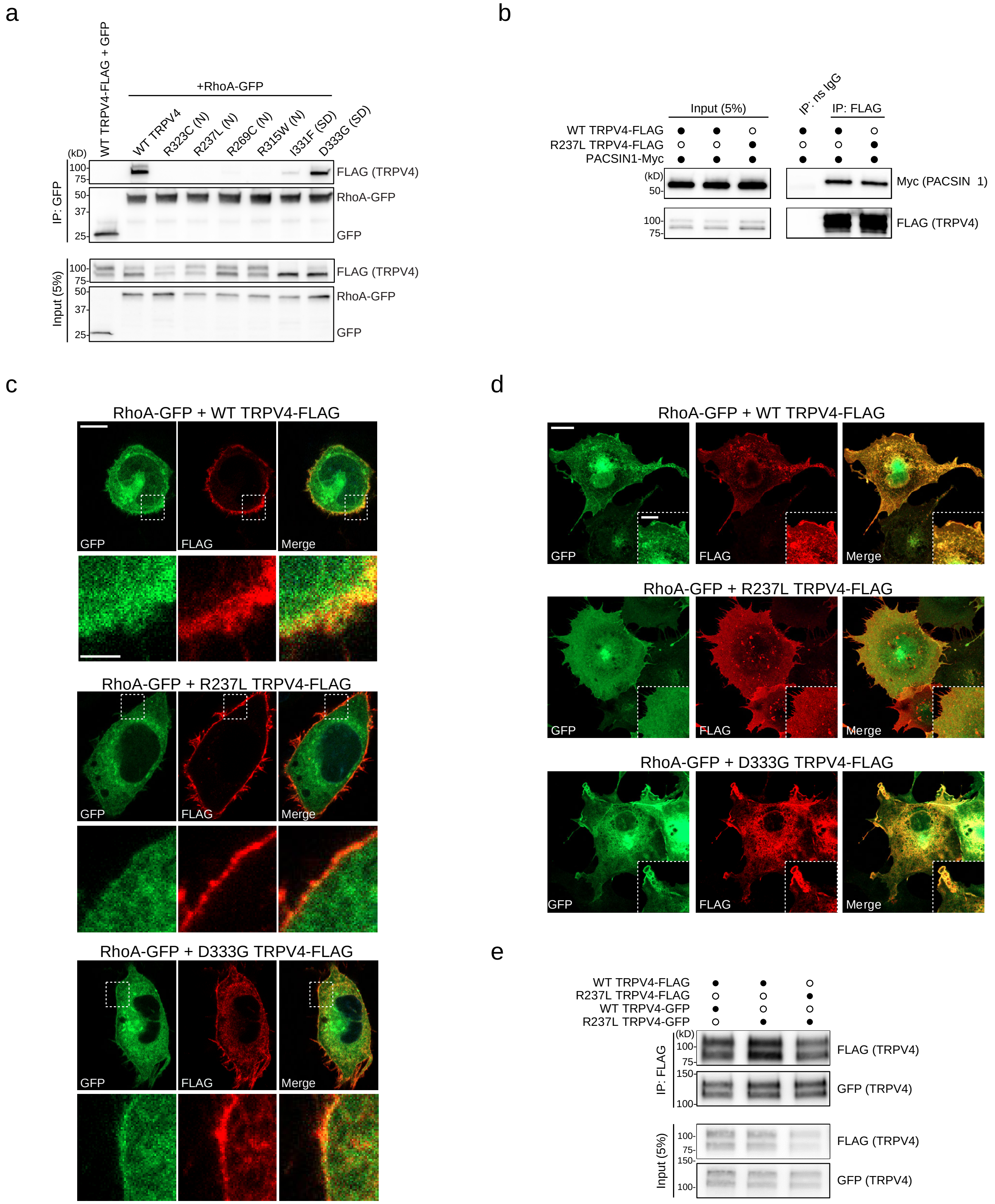

Supplementary Figure 2. TRPV4-RhoA interactions are disrupted by neuropathy mutations but not skeletal dysplasia mutations

(a) MN-1 cells were transfected with TRPV4-FLAG with neuropathy-causing mutations (N; R232C, R237L, R269C, R315W) or skeletal dysplasia-causing mutations (SD; I331F, D333G) and RhoA-GFP in the absence of TRPV4 antagonist, then subjected to immunoprecipitation with anti-GFP antibody. Neuropathy mutations, but not skeletal dysplasia mutations, disrupt interaction of TRPV4 and RhoA. Representative blot from two independent experiments.

(b) HEK293T cells were transfected with PACSIN1-Myc and WT or R237L TRPV4-FLAG in the presence of HC067 (0.5 µM), then subjected to immunoprecipitation with anti-FLAG antibody or non-specific IgG. Both WT and R237L TRPV4 co-immunoprecipitate with PACSIN1. Representative blot from at least three independent experiments.

(c) Immunofluorescence images of MN-1 cells transfected with TRPV4-FLAG and RhoA-GFP demonstrates TRPV4 and RhoA co-localization at the plasma membrane that is disrupted in neuropathy mutant TRPV4 (R237L) but preserved with skeletal dysplasia mutant TRPV4 (D333G). Representative image from three independent experiments. Scale bars, 10 µm and 2.5 µm for inset.

(d) Immunofluorescence images of COS7 cells transfected with TRPV4-FLAG and RhoA-GFP demonstrate TRPV4 and RhoA co-localization at the plasma membrane that is disrupted in neuropathy mutant TRPV4 (R237L), but preserved with skeletal dysplasia mutant TRPV4 (D333G). Representative image from two independent experiments. Scale bars, 20 µm and 10 µm for inset.

(e) MN-1 cells were transfected with WT or neuropathy mutant TRPV4-FLAG and/or TRPV4-GFP and subjected to immunoprecipitation with FLAG antibody. Both WT and neuropathy mutant TRPV4 can be co-immunoprecipitated, indicating that they are capable of interacting to form heterotetrameric channels. Blot image from one independent experiment.

Supplementary Figure 3

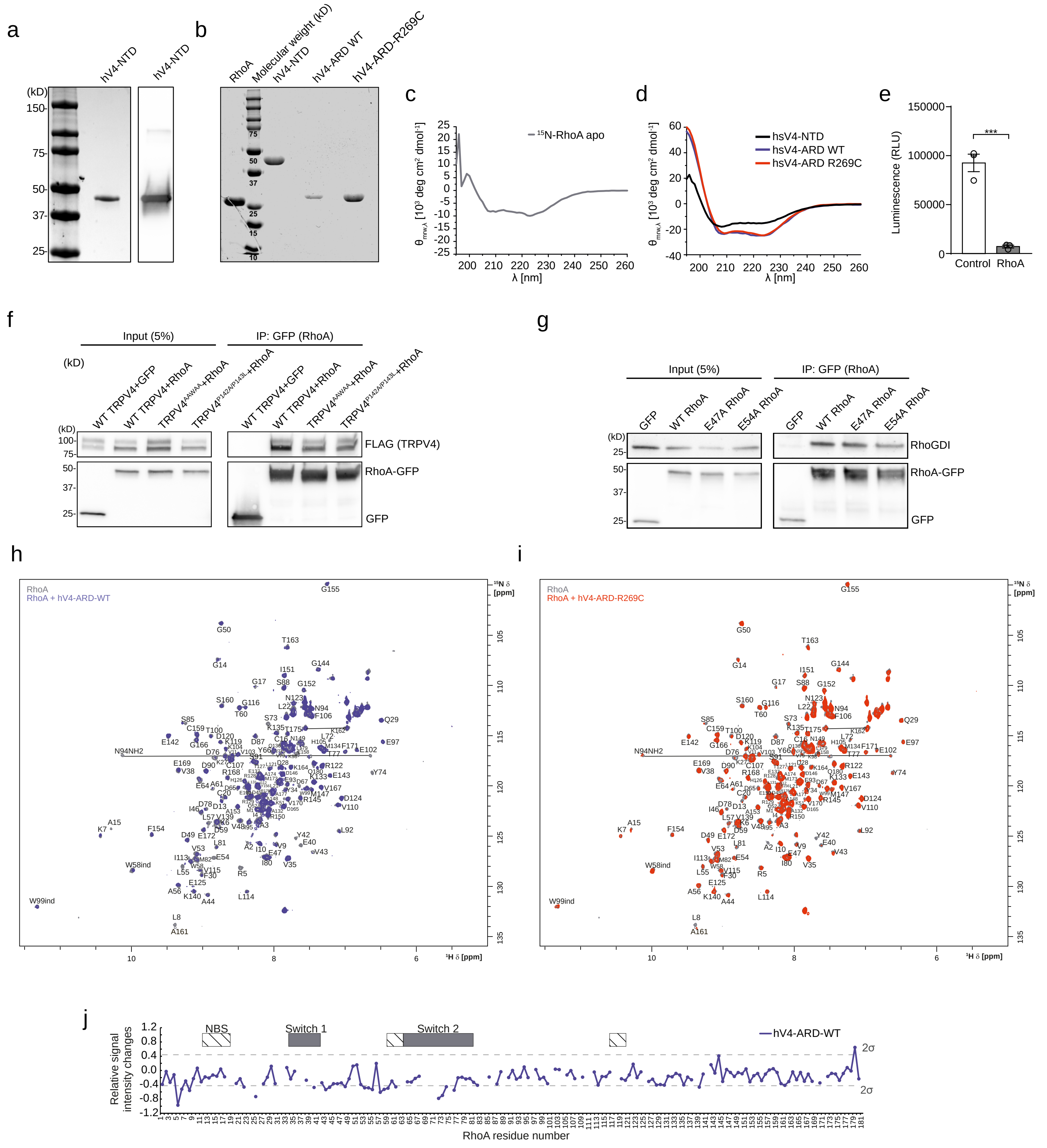

Supplementary Figure 3. Purified RhoA directly interacts with the TRPV4-ARD
(a) Coomassie stain (left) and western blot (right) of purified hV4-NTD (residues 2-397) with N-terminal specific TRPV4 antibody. Image from one independent experiment.
(b) Coomassie stain of purified human RhoA, hV4-NTD (residues 2-397), and WT and R269C mutant hV4-ARD (residues 148-397). Image from one independent experiment.
(c) Circular dichroism (CD) spectrum of human isotope-labeled ^{15}N-RhoA confirms properly folded protein.
(d) CD spectra of hV4-NTD, hV4-ARD-WT, and hV4-ARD-R269C confirms properly folded protein. As observed previously, both hV4-ARD-WT and hV4-ARD-R269C show predominantly an α-helical structure and a significant unstructured contribution in the hV4-NTD preceding the ARD[1,2].
(e) Intrinsic GTPase activity of purified ^{15}N-labeled RhoA was determined via the GTPase-Glo Assay. GTPase activity is inversely correlated to measured luminescence intensity. Paired two-tailed t test, $n = 3$ independent measurements from 4 technical replicates, ***$p = 0.0007$. Data are presented as mean values +/- SEM.
(f) MN-1 cells transfected with TRPV4-FLAG WT, ^{121}AAWAA^{125}, or P142A/P143L and GFP or RhoA-GFP were subjected to immunoprecipitation with anti-GFP antibody. TRPV4 binding to RhoA is preserved with mutation of the PI(4,5)P_2 binding domain (^{121}AAWAA^{125}) and proline-rich domain (P142A/P143L). Representative blot from three independent experiments.
(g) MN-1 cells transfected with RhoA-GFP WT, E47A, or E54A were subjected to immunoprecipitation with anti-GFP antibody. Interaction of RhoA with RhoGDI is preserved in E47A and E54A mutants. Representative blot from three independent experiments.
(h-i) 2D ^{1}H-^{15}N-NMR spectrum of ^{15}N-RhoA: Overlay of spectra of ^{1}H-^{15}N RhoA 2D HSCQs of ^{15}N-RhoA on its own (grey) confirms presence of a well-folded protein in the GDP-bound state. In the presence of hV4-ARD-WT (blue, f), significant line broadening is observed indicative of the formation of a high molecular weight complex. In contrast, addition of hV4-ARD-R269C (red, g) shows only minor effects on the RhoA spectrum. RhoA backbone NMR assignments were transferred from previously published data[3].
(**j**) Relative signal intensity changes of RhoA in the presence of unlabeled hsV4-ARD-WT. RhoA backbone NMR assignments were inferred from previously published data[3].

Supplementary Figure 4

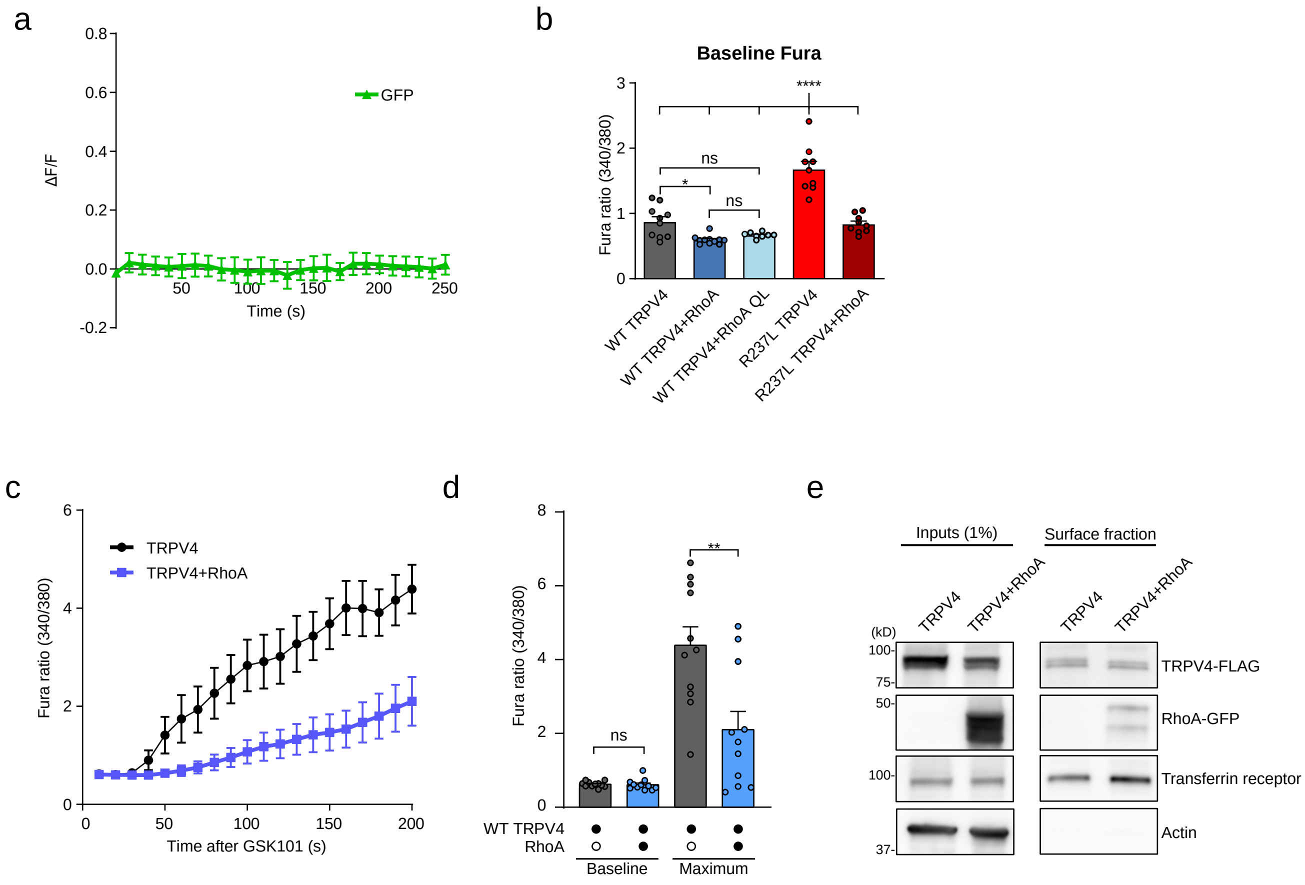

Supplementary Figure 4. RhoA inhibits TRPV4 channel activity, but does not alter TRPV4 surface expression

(a) MN-1 cells transfected with GFP alone were loaded with calcium indicator Fura-2 AM and treated with hypotonic saline (30 mM NaCl) at time 0. Transfected cells show no change in intracellular calcium in response to treatment with hypotonic saline, indicating few if any endogenous osmotically active ion channels in these cells. n = 6 independent coverslips per condition, each representing an average of 20-40 cells per coverslip.

(b) Quantification of baseline Fura ratio (340/380) from MN-1 cells shown in Figure 4a prior to stimulation with hypotonic saline. One-way ANOVA followed by Tukey's multiple comparisons test, n = 9 independent coverslips per condition, each representing an average of 20-40 cells per coverslip, *p = 0.0275, ****p < 0.0001.

(c) MN-1 cells were transfected with WT TRPV4-GFP alone or in combination with WT RhoA followed by measurement of intracellular calcium levels in response to treatment with the TRPV4 agonist GSK101 (15 nM) at time = 0. Data represents an average of n = 11 independent coverslips per condition, each representing an average of 20-40 cells per coverslip.

(d) Average baseline and maximum Fura ratios in MN-1 cells from (c). Unpaired two-tailed t test, **p = 0.0040.

(e) Surface biotinylation assay performed on HEK293T cells transfected with TRPV4-FLAG alone or in combination with RhoA-GFP showing that expression of RhoA does not affect the amount of TRPV4 expressed at the cell surface. Representative blot from two independent experiments.

Data are presented as mean values +/- SEM.

Supplementary Figure 5

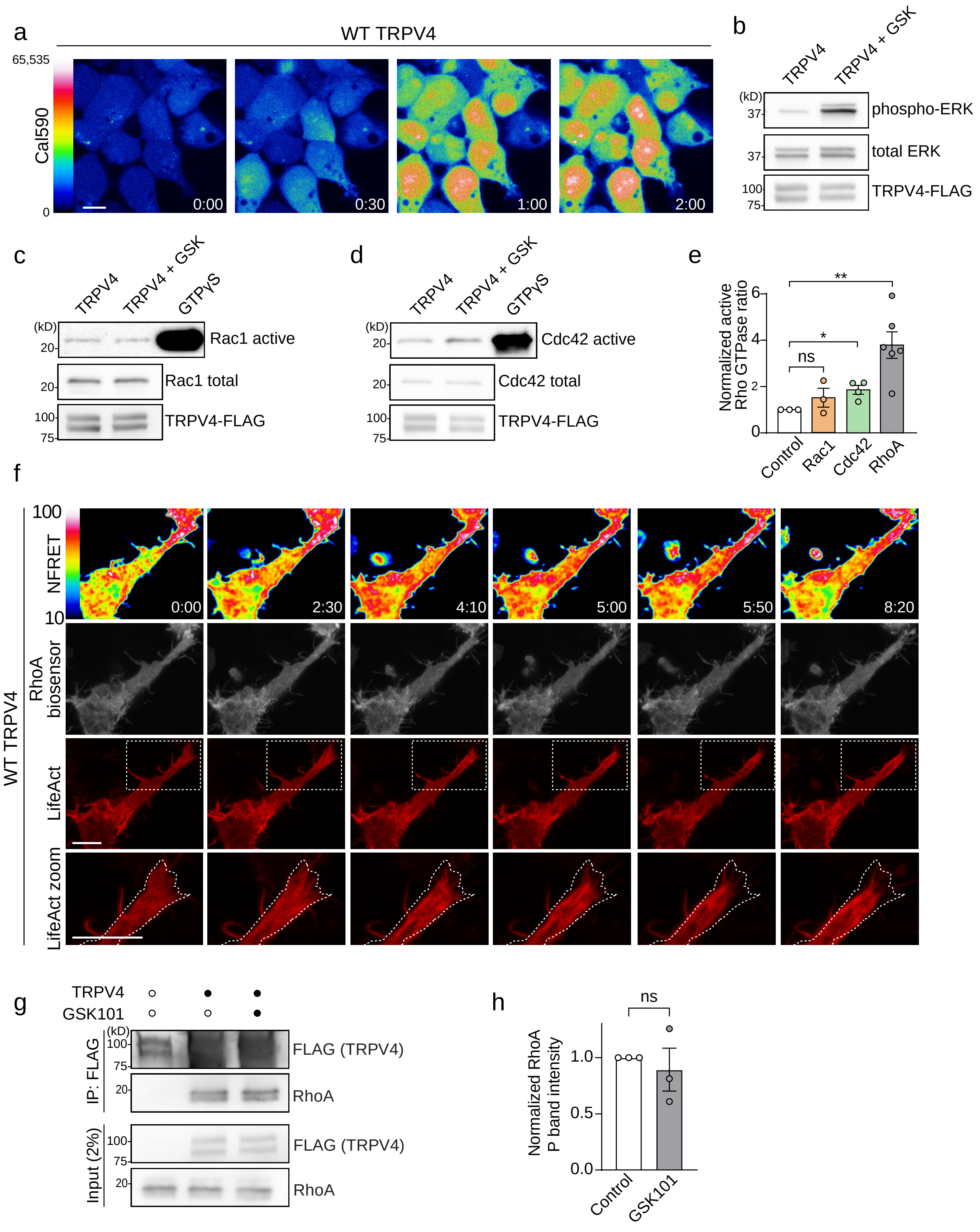

Supplementary Figure 5. TRPV4 channel activity leads to localized actin cytoskeletal changes and cell process retraction

(a) T-Rex-TRPV4^{WT} cells were induced with tetracycline (15 ng/ml) in the presence of HC067 for 16 h and then loaded with the calcium indicator Cal590. After removal of HC067, cells were treated with the TRPV4 agonist GSK101 (100 nM) and imaged every 10 sec for 2 min. TRPV4 agonist leads to increased intracellular calcium levels within 30 sec. Representative image from greater than 10 independent experiments. Scale bar indicates 10 µm.

(b) T-Rex-TRPV4^{WT} cells were induced with tetracycline (15 ng/ml) in the presence of HC067 for 16 h, followed by removal of HC067 and treatment with the TRPV4 agonist GSK101 (100 nM) for 5 min. GSK101 treatment leads to increased phospho-ERK, indicative of TRPV4 channel activation. Representative blot from three independent experiments.

(c-d) T-Rex-TRPV4^{WT} cells were treated as in (b) followed by analysis of Rac1 (c) and Cdc42 (d) activation using Rac1 and Cdc42 activation assay kits. Treatment with TRPV4 agonist leads to no significant change in Rac1 activation and a small but significant activation of Cdc42. Loading the reaction with GTPγS serves as a positive control for Rho GTPase activation.

(e) Quantification of densitometry of active Rac1, Cdc42, and RhoA band intensity divided by total Cdc42 or Rac1 band intensity, normalized to untreated condition; representative blots shown in (c) and (d). Paired two-tailed t test, n = 3 (Rac1), n= 4 (Cdc42), and n=6 (RhoA) independent experiments, *p = 0.0210, **p = 0.0045.

(f) T-Rex-TRPV4 cells were transfected with RhoA2G biosensor and LifeAct-mCherry followed by induction with tetracycline (15 ng/ml) in the presence of HC067 (0.5 µM) for 16 h. Upon HC067 removal, cells were treated with GSK101 (100 nM), and RhoA FRET and LifeAct were monitored by confocal microscopy for 10 min. Images show normalized FRET (NFRET, FRET signal divided by FRET donor intensity) (top), RhoA biosensor distribution by mVenus fluorescence (middle), and LifeAct-mCherry (bottom and zoom). Time series demonstrates localized stress fiber formation within an extended cell process, followed by retraction of the process. Scale bars, 10 µm for full image and inset.

(g) T-Rex-TRPV4^{WT} cells were induced in the presence of HC067 as described in (b) followed by treatment with GSK101 and immunoprecipitation of TRPV4. Treatment with TRPV4 agonist does not significantly alter TRPV4-RhoA interaction.

(h) Quantification of densitometry of RhoA IP band intensity divided by RhoA input band intensity; representative blot shown in (g). Paired two-tailed t test, n = 3 independent experiments.

Data are presented as mean values +/- SEM.

Supplementary Figure 6

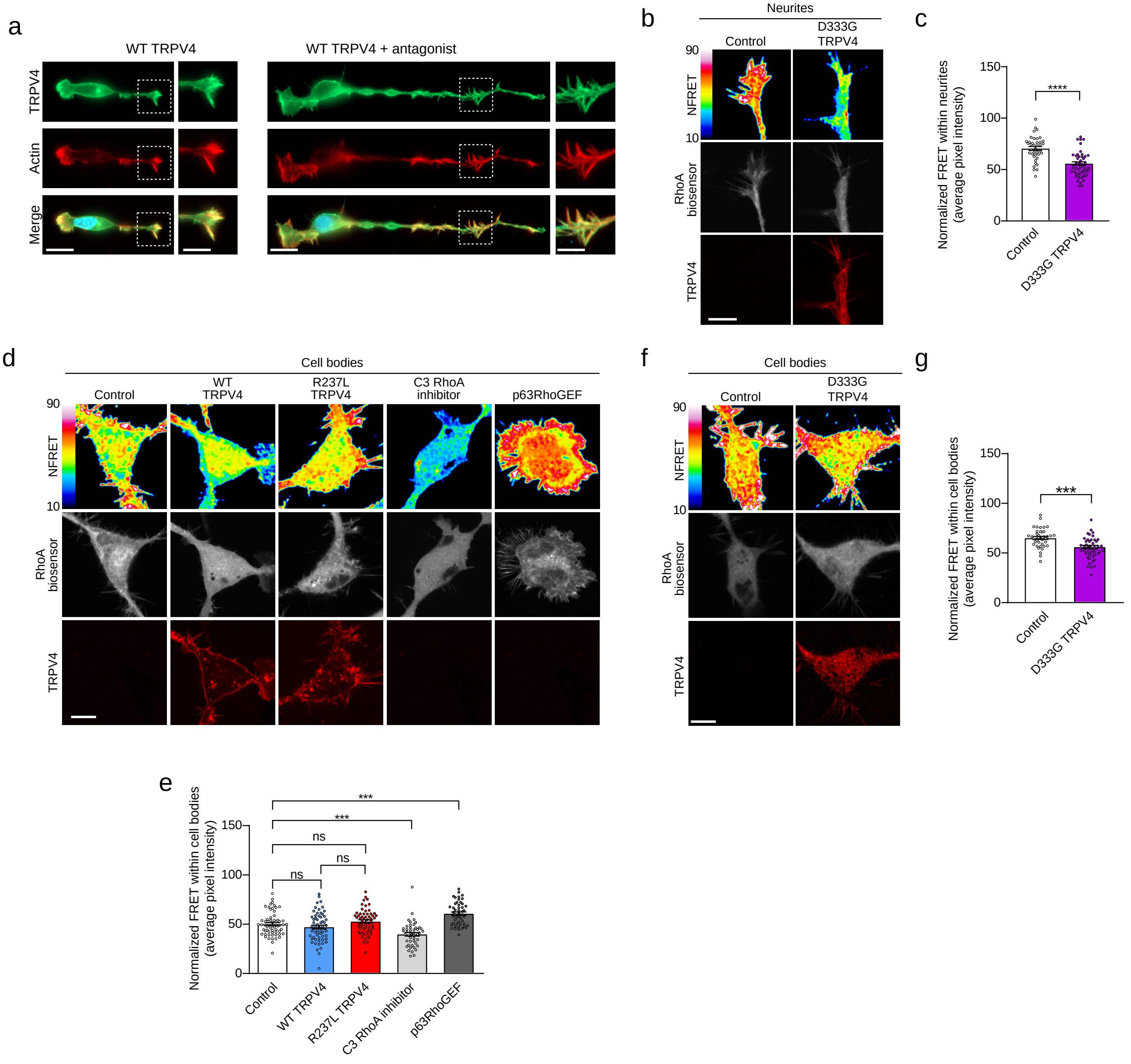

Supplementary Figure 6. TRPV4 localizes to actin-rich structures and preferentially inhibits RhoA within neurites

(a) Immunofluorescence images of MN-1 cells expressing TRPV4-FLAG in the presence or absence of TRPV4 antagonist HC067 (0.5 µM) show co-localization of TRPV4 with actin (phalloidin) within neurite-like structures. Representative image from at least 10 independent experiments. Scale bars, 20 µm.

(b) Representative images of MN-1 cells transfected with RhoA FRET biosensor alone or in combination with D333G-TRPV4-mScarlet in the presence of TRPV4 antagonist HC067 (0.5 µM). Images show RhoA NFRET (top), RhoA biosensor distribution by mVenus fluorescence (middle), and D333G-TRPV4- mScarlet (bottom) within neurites of MN-1 cells. D333G-TRPV4 results in decreased NFRET within neurites. Scale bar, 10 µm.

(c) Average NFRET within MN-1 cell neurites in experiments as shown in (b). Unpaired two-tailed t test, $n = 39$-49 neurites per condition, with one neurite chosen per cell body, from three independent experiments (control $n = 39$, D333G TRPV4 $n = 49$), ****$p < 0.0001$.

(d) Representative images of MN-1 cells transfected with RhoA FRET biosensor. Cells were either co-transfected with TRPV4 WT or R237L TRPV4-mScarlet in the presence of TRPV4 antagonist HC067 (0.5 µM) or p63RhoGEF-Myc or treated with C3 RhoA inhibitor for 2 h (1 µg/ml). Images show RhoA NFRET (top), RhoA biosensor distribution by mVenus fluorescence (middle), and TRPV4-mScarlet (bottom) within cell bodies of MN-1 cells. Scale bar, 10 µm.

(e) Average NFRET within MN-1 cell bodies demonstrating no significant differences among control, WT TRPV4, and R237L TRPV4-expressing cells in experiments as shown in (d). One-way ANOVA followed by Tukey's multiple comparisons test, $n = 47$-67 cell bodies per condition, from four independent experiments (control $n = 67$, WT TRPV4 $n = 63$, R237L $n = 58$, C3 $n = 47$, p63RhoGEF $n = 52$), ***$p = 0.0004$ (lane 1 vs 4), ***$p = 0.0002$ (lane 1 vs 5).

(f) Representative images of MN-1 cells transfected with RhoA FRET biosensor alone or in combination with TRPV4-D333G-mScarlet in the presence of TRPV4 antagonist HC067 (0.5 µM). Images show RhoA NFRET (top), RhoA biosensor distribution by mVenus fluorescence (middle), and TRPV4-D333G-mScarlet (bottom) within cell bodies of MN-1 cells. D333G-TRPV4 results in a small decrease in NFRET within cell bodies.

(g) Average NFRET within MN-1 cell bodies in experiments as shown in (f). Unpaired two-tailed t test, $n = 39$-49 cells per condition, from three independent experiments (control $n = 39$, D333G TRPV4 $n = 49$), ***$p = 0.0001$. Scale bars, 10 µm.

Data are presented as mean values +/- SEM.

Supplementary Table 1

**Protein**			Total MS/MS spectra (spectral count)			
**Gene**	Protein name	UniProtKB	WT	R237L	Ratio (WT/R237L)	Empty vector
**RHOA**	Transforming protein RhoA	P61586	112	66	1.7	43
**RPN2**	Dolichyl-diphosphooligosaccharide--protein glycosyltransferase subunit 2	P04844	97	51	1.9	15
**ATP2A2**	Sarcoplasmic/endoplasmic reticulum calcium ATPase 2	P16615	77	36	2.1	0
**MYBBP1A**	Myb-binding protein 1A	Q9BQG0	54	28	1.9	27
**DDOST**	Dolichyl-diphosphooligosaccharide--protein glycosyltransferase 48 kDa subunit	P39656	54	28	1.9	13
**ILF3**	Interleukin enhancer-binding factor 3	Q12906	37	0	-	0
**ZC3HAV1**	Zinc finger CCCH-type antiviral protein 1	Q7Z2W4	32	4	8.0	14
**GTPBP4**	Nucleolar GTP-binding protein 1	Q9BZE4	29	3	9.7	10
**USP9X**	Probable ubiquitin carboxyl-terminal hydrolase FAF-X	Q93008	29	12	2.4	0
**HDAC6**	Histone deacetylase 6	Q9UBN7	24	9	2.7	12
**SRPRB**	Signal recognition particle receptor subunit beta	Q9Y5M8	24	12	2.0	4
**DNAJA2**	DnaJ homolog subfamily A member 2	O60884	23	6	3.8	9
**DHCR7**	7-dehydrocholesterol reductase	Q9UBM7	23	6	3.8	6
**RFC1**	Replication factor C subunit 1	P35251	21	0	-	8
**B4GAT1**	Beta-1,4-glucuronyltransferase 1	O43505	21	2	10.5	0
**FAU**	40S ribosomal protein S30	P62861	20	0	-	0
**HDAC1**	Histone deacetylase 1	Q13547	20	8	2.5	0
**ESYT1**	Extended synaptotagmin-1	Q9BSJ8	20	9	2.2	0

Supplementary Table 1. Unbiased proteomics approach identifies putative TRPV4-interacting proteins

Results from an unbiased immunoprecipitation and liquid chromatography-mass spectrometry proteomic screen were stratified for relative enrichment in WT TRPV4 versus R237L TRPV4 and negative control (empty vector transfection). Results were filtered by setting a minimum threshold of 20 spectral counts and removing proteins that had less than 2-fold spectral count enrichment in WT TRPV4 as compared to the empty vector control. Further filtering to identify interactions that are potentially disrupted by the R237L neuropathy mutation was performed by removing proteins that had less than 1.5-fold spectral count enrichment in WT TRPV4 as compared to R237L TRPV4.

Primers used

Mutagenesis primers

TRPV4 M680K Fwd	tttaagctgaccatcggcaagggcgacctg
TRPV4 M680K Rev	caggtcgcccttgccgatggtcagcttaaa
TRPV4 R232C Fwd	ctcgatagtagatgtcacagaagggcgagttaatgaa
TRPV4 R232C Rev	ttcattaactcgcccttctgtgacatctactatcgag
TRPV4 R269C Fwd	gcccaggcctgtgggcgcttcttc
TRPV4 R269C Rev	gctggaagaagcgcccacaggcctg
TRPV4 I331F Fwd	ggtgttgtcagcaaaggccaccagcgcat
TRPV4 I331F Rev	atgcgctggtggcctttgctgacaacacc
TRPV4 D333G Fwd	ggtggccattgctggcaacacccgtgaga
TRPV4 D333G Rev	tctcacgggtgttgccagcaatggccacc
TRPV4 P142A/P143L Fwd	ttgaggatggggagcgcctgaggggcagg
TRPV4 P142A/P143L Rev	cctgcccctcaggcgctccccatcctcaa
TRPV4 121AAWAA125 Fwd	tcgtcaccactccagtgacaacgcggcgtgggcggcgaagatcatagagaagcagccg
TRPV4 121AAWAA125 Rev	cggctgcttctctatgatcttcgccgcccacgccgcgttgtcactggagtggtgacga
RhoA E47A Fwd	cctgctttccatccaccgcgatatctgccacatag
RhoA E47A Rev	ctatgtggcagatatcgcggtggatggaaagcagg
RhoA E54A Fwd	gtcccacaaagccaacgctacctgctttccatc
RhoA E54A Rev	gatggaaagcaggtagcgttggctttgtgggac
hV4-ARD-R269C Fwd	gcccaggcctgtgggcgcttcttc
hV4-ARD-R269C Rev	gctggaagaagcgcccacaggcctg

Generation of TRPV4 ARD expression plasmids

hV4-ARD_Insert Fwd	tttaagaaggagatatacatatgttcaaccggcctatcctct
hV4-ARD_Insert Rev	ggtgctcgagtgcggcctcaatgatgatggtgatgatgag
pET21_Backbone Fwd	ggccgcactcgagcaccaccaccaccaccactgagatc
pET21_Backbone Rev	atgtatatctccttcttaaagttaaacaaaattatttctagagggaa

Generation of T-Rex-TRPV4 cells

TRPV4 Trex Fwd	ttaagatatccagattaagcatggcggatt
TRPV4 Trex Rev	ttaagcggccgcctacttatcgtcatcgtcct

Generation of TRPV4-mScarlet

TRPV4 mScarlet Fwd	caagcttctcgagcatgcatatggtgagcaagggcgag
TRPV4 mScarlet Rev	gtcatcgtccttatagtcatctagatgcatgctcgacttgtacagctcgtccatgc

REFERENCES

1. Inada, H., Procko, E., Sotomayor, M. & Gaudet, R. Structural and biochemical consequences of disease-causing mutations in the ankyrin repeat domain of the human TRPV4 channel. *Biochemistry* **51**, 6195-6206 (2012).
2. Goretzki, B. *et al.* Structural Basis of TRPV4 N Terminus Interaction with Syndapin/PACSIN1-3 and PIP2. *Structure* **26**, 1583-1593 e1585 (2018).
3. Gasmi-Seabrook, G.M. *et al.* Real-time NMR study of guanine nucleotide exchange and activation of RhoA by PDZ-RhoGEF. *J Biol Chem* **285**, 5137-5145 (2010).
