# Supplementary material for: Neuropathy-causing TRPV4 mutations disrupt TRPV4-RhoA interactions and impair neurite extension
Source: Nat Commun. 2021 Mar 4;12:1444. doi: 10.1038/s41467-021-21699-y (PMC7933254; doi:10.1038/s41467-021-21699-y)
Supplement: Supplementary file 3 — Description of Additional Supplementary Files [file 41467_2021_21699_MOESM3_ESM.pdf]

Description of Additional Supplementary Files

Title: Supplementary Movie 1.

Description: WT TRPV4 ion channel stimulation increases RhoA activation Movie of T-Rex-TRPV4WT cells shown in Figure 5c. Images were acquired every 5 sec for 7 min following treatment with GSK101 (100 nM) at time = 0. Movie shows normalized FRET (NFRET, FRET signal divided by FRET donor intensity) (left) and RhoA biosensor distribution by mVenus fluorescence (right).

Title: Supplementary Movie 2.

Description: Neuropathy mutant TRPV4 ion channel stimulation increases RhoA activation Movie of T-Rex-TRPV4R269C cells shown in Figure 5c. Images were acquired every 5 sec for 7 min following treatment with GSK101 (100 nM) at time = 0. Movie shows normalized FRET (NFRET, FRET signal divided by FRET donor intensity) (left) and RhoA biosensor distribution by mVenus fluorescence (right). RhoA NFRET increased more robustly in cells expressing neuropathy mutant TRPV4 compared to cells expressing WT TRPV4.

Title: Supplementary Movie 3.

Description: TRPV4-mediated calcium influx precedes RhoA activation Movie of T-Rex-TRPV4R269C cells shown in Figure 5f. Images were acquired every 10 sec for 15 min following treatment with GSK101 (100 nM) at time = 0. Movie shows normalized FRET (NFRET, FRET signal divided by FRET donor intensity) (left), RhoA biosensor distribution by mVenus fluorescence (middle), and Cal590 (right). Intracellular calcium increase precedes RhoA activation by 30-60 sec.

Title: Supplementary Movie 4.

Description: TRPV4 ion channel stimulation leads to activation of RhoA and changes in the actin cytoskeleton Movie of T-Rex-TRPV4WT cells shown in Figure 5h. Images were acquired every 10 sec for 10 min following treatment with GSK101 (100 nM). Movie shows normalized FRET (NFRET, FRET signal divided by FRET donor intensity) (left), RhoA biosensor distribution by mVenus fluorescence (middle), and LifeAct-mCherry (right). Activation of RhoA results in actin stress fiber formation and cellular contraction.

Title: Supplementary Movie 5.

Description: TRPV4 ion channel stimulation leads to activation of RhoA, stress fiber formation, and cell process retraction Movie of T-Rex-TRPV4WT cells shown in Supplementary Figure 5f. Images were acquired every 10 sec for 9.5 min following treatment with GSK101 (100 nM). Movie shows

normalized FRET (NFRET, FRET signal divided by FRET donor intensity) (left), RhoA biosensor distribution by mVenus fluorescence (middle), and LifeAct-mCherry (right). Activation of RhoA results in actin stress fiber formation followed by cellular process retraction.
