# Supplementary material for: Neuropathy-causing TRPV4 mutations disrupt TRPV4-RhoA interactions and impair neurite extension
Source: Nat Commun. 2021 Mar 4;12:1444. doi: 10.1038/s41467-021-21699-y (PMC7933254; doi:10.1038/s41467-021-21699-y)
Supplement: Supplementary file 9 — Reporting Summary [file 41467_2021_21699_MOESM9_ESM.pdf]

nature research

Corresponding author(s): Charlotte Sumner, Brett McCray

Last updated by author(s): 1/29/2020

# Reporting Summary

Nature Research wishes to improve the reproducibility of the work that we publish. This form provides structure for consistency and transparency in reporting. For further information on Nature Research policies, see our Editorial Policies and the Editorial Policy Checklist.

## Statistics

For all statistical analyses, confirm that the following items are present in the figure legend, table legend, main text, or Methods section.

| n/a | Confirmed | |
|---|---|---|
| ☐ | ☒ | The exact sample size (*n*) for each experimental group/condition, given as a discrete number and unit of measurement |
| ☐ | ☒ | A statement on whether measurements were taken from distinct samples or whether the same sample was measured repeatedly |
| ☐ | ☒ | The statistical test(s) used AND whether they are one- or two-sided<br>*Only common tests should be described solely by name; describe more complex techniques in the Methods section.* |
| ☒ | ☐ | A description of all covariates tested |
| ☐ | ☒ | A description of any assumptions or corrections, such as tests of normality and adjustment for multiple comparisons |
| ☐ | ☒ | A full description of the statistical parameters including central tendency (e.g. means) or other basic estimates (e.g. regression coefficient) AND variation (e.g. standard deviation) or associated estimates of uncertainty (e.g. confidence intervals) |
| ☐ | ☒ | For null hypothesis testing, the test statistic (e.g. *F*, *t*, *r*) with confidence intervals, effect sizes, degrees of freedom and *P* value noted<br>*Give P values as exact values whenever suitable.* |
| ☒ | ☐ | For Bayesian analysis, information on the choice of priors and Markov chain Monte Carlo settings |
| ☒ | ☐ | For hierarchical and complex designs, identification of the appropriate level for tests and full reporting of outcomes |
| ☒ | ☐ | Estimates of effect sizes (e.g. Cohen's *d*, Pearson's *r*), indicating how they were calculated |

*Our web collection on statistics for biologists contains articles on many of the points above.*

## Software and code

Policy information about availability of computer code

| | |
|---|---|
| Data collection | Epifluorescence and confocal fluorescence microscopy images were acquired using ZEN software (Zeiss, v3.1). Ratiometric alcium imaging data was collected using AxioVision software (Zeiss, v4). |
| Data analysis | Liquid chromatography-mass spectrometry data was analyzed using Proteome Discoverer (Thermo Fisher Scientific, v1.4) and using Mascot (Matrix Science, v2.5.1). NMR HSCQs were processed using TopSpin (Bruker, v3.2). RhoA FRET images were analyzed using the PIXFRET plugin for FIJI (NIH, v1.53). Statistical analysis was performed using Prism (Graphpad, v8). |

For manuscripts utilizing custom algorithms or software that are central to the research but not yet described in published literature, software must be made available to editors and reviewers. We strongly encourage code deposition in a community repository (e.g. GitHub). See the Nature Research guidelines for submitting code & software for further information.

## Data

Policy information about availability of data

All manuscripts must include a data availability statement. This statement should provide the following information, where applicable:

- Accession codes, unique identifiers, or web links for publicly available datasets
- A list of figures that have associated raw data
- A description of any restrictions on data availability

All data supporting the findings of this study and unique biological materials used in this study are available from the corresponding authors upon reasonable request. The numerical source data for all main figures and supplementary figures is provided in the Source Data file. Uncropped images of all blots from main figures and supplementary figures are also provided in the Source Data file. The mass spectrometry proteomics data have been deposited to the ProteomeXchange Consortium via the PRIDE partner repository with the dataset identifier PXD023758 and 10.6019/PXD023758; http://www.ebi.ac.uk/pride/archive/projects/PXD023758.

# Field-specific reporting

Please select the one below that is the best fit for your research. If you are not sure, read the appropriate sections before making your selection.

- [x] Life sciences
- [ ] Behavioural & social sciences
- [ ] Ecological, evolutionary & environmental sciences

For a reference copy of the document with all sections, see nature.com/documents/nr-reporting-summary-flat.pdf

# Life sciences study design

All studies must disclose on these points even when the disclosure is negative.

| | |
|---|---|
| Sample size | For ratiometric calcium imaging, sample size was predetermined to be at least n=9 from three experimental replicates obtained from three independent experiments on different days. This predetermination was based on results from prior unrelated experiments demonstrating that this sample size yielded standard error of the mean that was less than 10% of the mean. For MN-1 neurite length quantification, sample size was predetermined to be at least n=3 based on previous experiments demonstrating robust reproducibility and consistency of the data such that standard error of the mean that was less than 10% of the mean. In many cases, a greater n was used due to inclusion of repeated controls for many of the experimental replicates. Sample sizes were not predetermined other experiments. In these cases, sample sizes were deemed sufficient once the standard error of the mean was approximately 10% of the mean. |
| Data exclusions | No data were excluded |
| Replication | No experiments shown failed to replicated. All experiments were replicated by at least three independent experiments performed on different days. |
| Randomization | For ratiometric calcium imaging, the center of each coverslip was imaged. For live imaging experiments, the experimental conditions were recorded in an alternating fashion (i.e. condition 1, condition 2, condition 3, condition 1, condition 2, condition 3) on a given day, and the order was then changed on subsequent days. For image acquisition for quantification of neurite length, five fields per coverslip were chosen using a blinded system in which an image was taken in four quadrants and in the middle of the coverslip. For neurite FRET image acquisition, cells expressing roughly equivalent levels of the RhoA biosensor were chosen at random. For Drosophila larval dissections, subjects were selected at random. |
| Blinding | For quantification of MN-1 neurite length and larval neurite Sholl analysis, images were blinded. Blinding was performed by assigning each image a random number, renaming the files with that number, quantifying the numbered files, and then unblinding based on the image number after quantification was completed. All other experiments were not blinded as it was deemed unnecessary or not feasible. Specifically, western blot densitometry blinding was deemed unnecessary as standardized approaches for band identification and quantification were employed. Blinding for calcium imaging and NFRET quantification was deemed unnecessary as generation of cell ROIs and subsequent quantification was performed in a standardized manner with most steps being automated. |

# Reporting for specific materials, systems and methods

We require information from authors about some types of materials, experimental systems and methods used in many studies. Here, indicate whether each material, system or method listed is relevant to your study. If you are not sure if a list item applies to your research, read the appropriate section before selecting a response.

## Materials & experimental systems

| n/a | Involved in the study | |
|---|---|---|
| ☐ | ☒ | Antibodies |
| ☐ | ☒ | Eukaryotic cell lines |
| ☒ | ☐ | Palaeontology and archaeology |
| ☐ | ☒ | Animals and other organisms |
| ☒ | ☐ | Human research participants |
| ☒ | ☐ | Clinical data |
| ☒ | ☐ | Dual use research of concern |

## Methods

| n/a | Involved in the study | |
|---|---|---|
| ☒ | ☐ | ChIP-seq |
| ☒ | ☐ | Flow cytometry |
| ☒ | ☐ | MRI-based neuroimaging |

## Antibodies

| | |
|---|---|
| Antibodies used | Primary antibodies used were rabbit anti-FLAG (WB 1:1000, Cell Signaling Technology, 2368), mouse anti-FLAG (WB 1:1000, IF 1:1500, Cell Signaling Technology, 8146), rabbit anti-GFP (WB 1:1000, Cell Signaling Technology, 2555), rabbit anti-Myc (WB 1:1000, Cell Signaling Technology, 2272), mouse anti-Myc (WB 1:1000, IF 1:500, IP 5μg/ml, Cell Signaling Technology, 2276), rabbit anti-RhoA (WB 1:1000, Cell Signaling Technology, 2117), mouse Rac1/2/3 (WB 1:1000, Cell Signaling Technology, 2465), and mouse Cdc42 (WB 1:1000, Cell Signaling Technology, 2462), rabbit anti-His (WB 1:1000, Cell Signaling Technology, 2365), rabbit anti-β-actin (WB 1:1000, Cell Signaling Technology, 4967), rabbit anti-RhoGDI (WB 1:1000, Cell Signaling Technology, 2564), rabbit phospho-Thr202/Tyr204 ERK1/2 (WB 1:1000, Cell Signaling Technology 9101), rabbit ERK1/2 (WB 1:1000, Cell Signaling Technology, 9102), mouse anti-FLAG M2 (IP 5μg/ml, Sigma-Aldrich, F1804), rabbit anti-GFP (WB 1:1000, Thermo Fisher Scientific, A-11122), mouse anti-GFP (IP 5μg/ml, |

| | |
|---|---|
| | Thermo Fisher Scientific, A-11120), rabbit TRPV4 (WB 1:1000, Cosmo Bio USA, KAL-KM119), rabbit anti-TRPV4 (WB 1:1000, IP 5µg/ml, Abcam, ab39260), non-specific rabbit IgG (IP 5µg/ml, Cell Signaling Technology, 3900), and anti-mouse RhoA (WB 1:1000, Thermo Fisher Scientific, 1B3-4A10). Secondary antibodies used were HRP-conjugated monoclonal mouse anti-rabbit IgG, light chain specific (1:50,000, Jackson ImmunoResearch, 211-032-171), HRP-conjugated goat anti-mouse IgG, light chain specific (1:25,000, Jackson ImmunoResearch, 211-032-174), Alexa Fluor 488 goat anti-rabbit (1:1000, Thermo Fisher Scientific, A-11034), Alexa Fluor 488 goat anti-mouse (1:1000, Thermo Fisher Scientific, A-11029), Alexa Fluor 568 goat anti-rabbit (1:1000, Thermo Fisher Scientific, A-11011), and Alexa Fluor 555 goat anti-mouse (1:1000, Thermo Fisher Scientific, A-21422). Reagents used include C3 transferase (Cytoskeleton, Inc., CT03), HC067047 (Sigma-Aldrich, SML0143), GSK1016790A (Sigma-Aldrich, G0798), GDP (Sigma-Aldrich, 20-177), GTPγS (Sigma-Aldrich, 20-176). |
| Validation | All antibodies obtained from Cell Signaling Technology, Sigma-Aldrich, and Thermo Fisher Scientific have been validated by the company and are widely used in the literature. Specific information for each antibody used can be found on the manufacturer website by searching for the product number referenced above (https://www.cellsignal.com; https://www.sigmaaldrich.com; https://www.thermofisher.com). Antibodies to epitode tags (GFP, Myc, FLAG, and His) were further validated by our lab by western blot and immunofluorescence using untransfected and epitope-tag transfected cells as negative and positive controls, respectively. Rabbit TRPV4 (Cosmo and Abcam) were validated by performing western blots of lysates from cell lines that do not express TRPV4 as well as mouse TRPV4 knockout tissue. |

## Eukaryotic cell lines

Policy information about cell lines

| | |
|---|---|
| Cell line source(s) | MN-1 cells were obtained from Dr. Kenneth Fischbeck at the NIH. HEK293T cells were obtained from ATCC (CRL-11268). T-Rex-TRPV4WT and T-Rex-TRPV4R269C cells were generated as described in Methods. COS7 cells were obtained from ATCC (CRL-1651). |
| Authentication | MN-1, COS7, and HEK293T cells were not authenticated. T-Rex-TRPV4WT and T-Rex-TRPV4R269C cells were tested to confirm low background expression by western blot and RT-PCR and tested for inducibility using escalating doses of tetracycline followed by western blot. |
| Mycoplasma contamination | Cell lines were not tested for mycoplasma contamination. |
| Commonly misidentified lines (See ICLAC register) | No commonly misidentified cell lines were used. |

## Animals and other organisms

Policy information about studies involving animals; ARRIVE guidelines recommended for reporting animal research

| | |
|---|---|
| Laboratory animals | Transgenic Drosophila, male and female, where used from wandering third instar larval stage (larval day 4). Wild type male C57BL6/J mice, aged 3-9 weeks, were used to harvest choroid plexus epithelium. |
| Wild animals | No wild animals were used. |
| Field-collected samples | No field-collected samples were used. |
| Ethics oversight | Mice were maintained according to protocols and ethical regulations approved by the Johns Hopkins University Institutional Animal Care and Use Committee. |

Note that full information on the approval of the study protocol must also be provided in the manuscript.
